# How Can We Find Out What Indigenous Children and Their Families Need to Manage Weight? Lessons from Formative Nutrition Intervention Research with First Australians

**DOI:** 10.3390/nu15234982

**Published:** 2023-12-01

**Authors:** Lauren T. Williams, Mari Somerville, Fiona Wright, Heidi Atkins, Ayala Rogany, Kristie L. Bell, Lisa Vincze

**Affiliations:** 1 Menzies Health Institute of Queensland, Griffith University, Gold Coast, QLD 4222, Australia; lauren.williams@griffith.edu.au; 2 School of Health Sciences and Social Work, Griffith University, Gold Coast, QLD 4222, Australia; mari.somerville@dal.ca (M.S.); fiona.wright@griffith.edu.au (F.W.); 3 GUMURRII Student Success Unit, Griffith University, Gold Coast, QLD 4222, Australia; 4 Queensland Child and Youth Clinical Network, Clinical Excellence Queensland, Queensland Government, Brisbane, QLD 4006, Australia; heidi.atkins@health.qld.gov.au; 5 Reform Office, Strategy, Policy and Reform Division, Queensland Government, Brisbane, QLD 4000, Australia; 6 Dietetics and Food Services, Children’s Health Queensland Hospital and Health Service, South Brisbane, QLD 4001, Australia; ayala.rogany@health.qld.gov.au (A.R.); kristie.bell@health.qld.gov.au (K.L.B.)

**Keywords:** culturally tailored, participant, action research, qualitative, First Nations peoples, Indigenous, Aboriginal and Torres Strait Islander, decolonized research

## Abstract

In Australia, Indigenous children have rates of overweight and obesity 1.5 times those of non-Indigenous children. Culturally safe and effective nutrition interventions are needed for this group. This paper aims to describe a Community-based Participatory Action Research (CPAR) approach to designing formative nutrition intervention research with First Australian children and their families and to reflect on the challenges arising from this process. After obtaining ethical approvals, a Steering Committee (SC), including nine Aboriginal and Torres Strait Islander people experienced in delivering or receiving health care, was established as a project governance body to develop culturally safe project materials and methods. The Indigenous research method of yarning circles was chosen by the SC for the community consultation, and the First Australian SC members were trained to collect the data. They liaised with community organizations to recruit yarning circle participants. Individual interviews conducted by an Aboriginal research assistant replaced yarning circles due to the COVID-19 pandemic lockdowns. While the CPAR approach to formative research was successful, the pandemic and other factors tripled the study duration. To authentically, ethically and safely engage First Australians in research, researchers need to decolonize their methodological approach, and funding bodies need to allow adequate time and resources for the process.

## 1. Introduction

Indigenous is a term applied to the first known inhabitants of a country. Aboriginal and Torres Strait Islander peoples were the first inhabitants of Australia, and the term First Australians will be used in this paper to respectfully refer to the Indigenous people of Australia [1]. Through their connection to country and knowledge of flora and fauna, the traditional diets of First Australians were highly nutritious [2], and they lived healthy lifestyles without obesity or chronic disease prior to the invasion that commenced in the 18th century [3]. Colonization resulted in dispossession of the land, preventing access to traditional foods, which were replaced with rations high in fat, salt, sugar and empty calories [3]. Over subsequent generations, these imposed living conditions resulted in the emergence of chronic diseases in this population, which now has a high prevalence of heart disease, diabetes and obesity [4]. The most recent national survey of the health of the First Australian population conducted in 2018–2019 found that 46% had at least one chronic condition, shortening their lifespan in comparison to non-Indigenous Australians [4]. These chronic diseases have become so prevalent that they are evident in children and young people [4].

Young people comprise more than half of the First Australian population, 52.3% of which is younger than 25 years of age [5]. The 2018–2019 health survey of First Australians showed a high and rising prevalence of overweight and obesity in the population, estimated to contribute 15% to their shortened lifespan [4]. Overweight and obesity in children 2–14 years is higher in prevalence (24% overweight, 13% obese, 37% total) [4] than for non-Indigenous children of the same age (16.4% overweight, 8.0% obese, 24.4% total) [6]. While the prevalence of overweight and obesity remained stable in non-Indigenous children compared with the previous national survey [6], in First Australian children, the prevalence increased from 30% to 37% in just six years [4]. First Australian children appear to be at a higher risk of weight gain than non-Indigenous children of the same age due to nutritional factors, with a much higher proportion of the population reporting to consume sugar-sweetened beverages at least weekly (58.8% vs. 16.5%) and a slightly lower proportion consuming the recommended number of fruit and vegetable serves (6.2% vs. 9.2%) [4,7]. While these statistics imply a simple causation of overweight and obesity, the factors influencing dietary intake are complex and involve the social determinants of health [8].

The disparity in health status between Indigenous and non-Indigenous populations requires urgent attention, and there are international, national and state initiatives aimed at ‘Closing the Gap’ [8,9,10]. At the international level, the World Health Organization considers the social determinants of health in arguing the need to focus on children and young people as the best chance for reducing health inequities within a generation [8]. Progress toward equality for First Australians has been slow but will hopefully be improved by the partnership formed in 2020 between all levels of government and a coalition of Aboriginal and Torres Strait Islander peak organizations to develop the National Agreement on Closing the Gap [11]. The initiative highlights the need for government institutions to be culturally safe and responsive to the needs of First Australians [11], which includes the delivery of health care. The health system has traditionally provided a structural barrier to health equity for First Australians, who have developed a distrust of non-Indigenous health systems through lived experiences of systemic and interpersonal racism [12,13]. If health equity is to be achieved, preventive and treatment-oriented health interventions in the form of services and programs must be culturally safe and tailored to the needs of First Australians [14].

Nutritional interventions are planned activities aimed at changing an aspect of dietary intake in order to improve the health of an individual or group [15]. The intervention planning process ideally follows formative research conducted to explore the preferences and needs of the target group, with the findings used to guide the intervention planning [16]; however, the formative research stage is often missing from implementation research [17]. Formative research is seen as particularly important when interventions are being planned for Indigenous groups, given that research is frequently conducted by those outside the community [16]. Our systematic scoping review of the literature on nutrition interventions with Indigenous populations around the world found that while 52 of the 66 included interventions cited formative research, only 28 reported conducting that research with the relevant Indigenous community [18]. For those research teams that do plan to conduct formative research, there is a risk that non-Indigenous researchers lack the lived experience to design a research protocol to explore the perceptions and needs of Indigenous communities. A systematic review of 26 nutrition-related interventions in First Australians found that only half of the studies achieved moderate or strong community engagement [19]. A scoping review of 21 nutrition interventions for cardiovascular disease with First Australians found that while most of the interventions achieved community engagement and capacity strengthening, few reported taking a strengths-based approach or adopting Indigenous research paradigms and governance [20]. An approach that deeply and authentically involves the community concerned in the research process would clearly be advantageous.

A community-based participatory action research (CPAR) approach, as the name implies, involves **participation** by members of the **community** in the **research** process aimed at achieving change (**action**) [21]. The emphasis is on respectful engagement with the community throughout a collaborative process [22]. Together with Action Research and Participatory Action Research, CPAR is part of a group of approaches that arose from the social sciences and are increasingly applied in public health [23]. The approach is seen as particularly relevant in research with Indigenous communities due to the attempt to reduce the colonizing effects of traditional research through community participation [23,24]. The aim of this paper is to describe the ways in which members of the First Australian community were involved in the design and collection of formative research assessing the weight management needs of their children. A secondary aim is to consider the lessons learned from the advantages and limitations of this approach. While other types of data were collected as part of the broader formative research (health utilization data, interviews with non-Indigenous health care providers), these methods are not described here to maintain the focus on strategies for engagement with the First Australian community.

## 2. Materials and Methods

### 2.1. Study Design, Setting and Population

This formative research used a qualitative design within a CPAR approach [21,24,25] to explore the felt needs of First Australians for a pediatric weight management intervention. The formative research was part of a broader study initially funded for an 18-month period. While the request for the research did not arise directly from the community, it did arise from a concern by health care providers that existing pediatric weight management services were designed and delivered by non-Indigenous health professionals and were under-utilized by First Australians. The project was designed to develop interventions that would be culturally safe and acceptable to the community. The research management team set the following three objectives to make the research process consistent with a CPAR approach and with national guidelines for ethical research with Aboriginal and Torres Strait Islander communities [26]:

1. Empower the First Australian community to investigate the problem and explore possible solutions.
2. Develop the research capacity (skills in data collection) of First Australians.
3. Ensure cultural safety for First Australian research participants.

The intention was to take a ‘strengths-based’, rather than a ‘deficits’, approach [27]. Community engagement was important in the design of this formative research to minimize the risk imposed by the ‘whiteness’ of members of the research team [12,13] and to empower the community to investigate the problem and explore possible solutions. Ethical approval to conduct the research was obtained from the relevant agencies.The ethical approach was consistent with national guidelines for ethical research with Aboriginal and Torres Strait Islander communities [26]. This paper is reported according to the consolidated criteria for strengthening reporting of health research involving Indigenous peoples (CONSIDER) statement [28]. The report draws on the minutes of meetings by the research management team and the Steering Committee and the reflections of researchers.

The Australian state of Queensland has one of the largest First Australian populations in the country. In the 2021 national census, 237,000 people in the state of Queensland identified as First Australians, comprising 4.6% of the Queensland population compared with the national average of 3.2% [5]. Previous research had established that, while two-thirds of the Aboriginal and Torres Strait Islander health workforce in Queensland saw childhood obesity as a priority issue for their First Australian communities, culturally appropriate interventions to support weight management were lacking [29]. Thus, it was important to involve Aboriginal and Torres Strait Islander health workers in conducting the formative research needed to design a culturally appropriate intervention. The population for the formative research was Aboriginal and Torres Strait Islander children and their families living in south–east Queensland, an area chosen for the location of the research team and population density.

The research management team was drawn from Griffith University and Children’s Health Queensland, who operated together under a research collaborative agreement. At the time of obtaining grant funding and initial ethical approval, the research management team consisted of four professionals: two public health nutrition academics with some experience in health care delivery to First Australian communities; the Research Manager for Children’s Health Queensland, who had experience working with Aboriginal and Torres Strait Islander health workers; and the Principal Policy Officer for the Queensland Child and Youth Clinical Network, who managed an extensive network of Aboriginal and Torres Strait Islander health workers. These four researchers were non-Indigenous and had not previously completed a formal research project with the First Australian community. In an attempt to address this deficit, the researchers completed cultural sensitivity training, consulted with academics from the First Peoples Unit at Griffith University, undertook self-education through attending an Indigenous Research and Knowledges seminar series and recruited other researchers to the research management team to broaden team capacity and capability. An Aboriginal woman, a descendant of the Kamilaroi tribe, was employed as a research assistant. The research management team met online at least fortnightly to discuss the data collection, with meeting minutes stored on a secure research drive. This team held responsibility for the administrative and reporting aspects of grant management.

### 2.2. Community Engagement Guiding Formative Research Design: The Steering Committee

**Figure 1:**
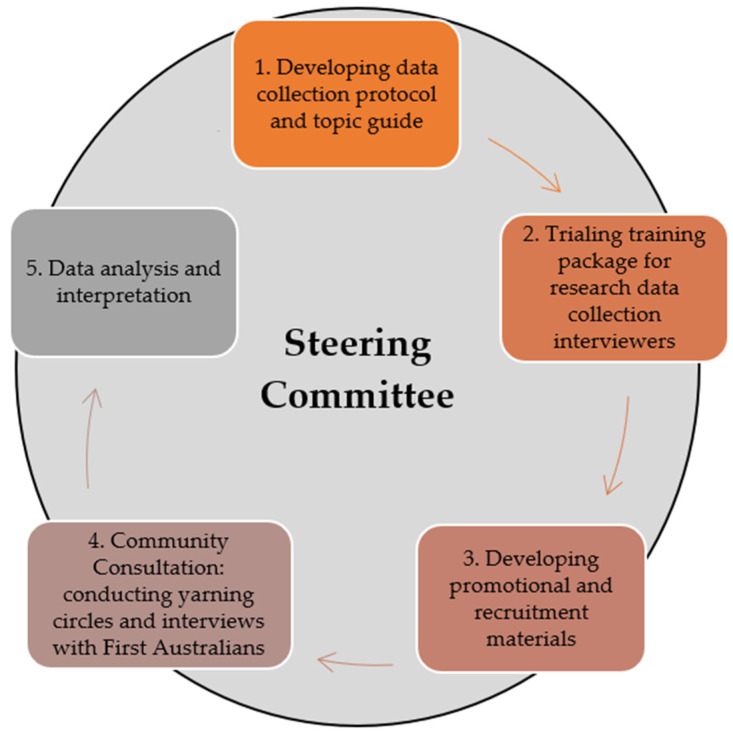
The central role of the Steering Committee in guiding formative research on the felt needs of First Australians.

Our previous scoping review of nutrition interventions with Indigenous groups found that while most interventions used more than one cultural tailoring strategy, fewer used strategies that involved constituents at a deep level [18]. Therefore, we aimed for this formative research to incorporate a variety of cultural tailoring strategies and to have at its heart ‘deep’ structures that addressed ‘the cultural, social, historical, environmental and psychological forces that influence the target health behavior’ [30] (p.12). The main community engagement strategy guiding the formative research was the establishment of a Steering Committee (SC). Figure 1 illustrates the central role of the SC in the stages of the formative research project.

The research project SC was established as a governance structure for the research, adapting the method used by Canuto and colleagues, who established a local advisory committee to guide the planning for their intervention with a community of First Australian adult women [31]. The SC was convened at the beginning of the research process and operated under a Terms of Reference document. The SC was tasked with providing strategic guidance and advice on culturally safe research protocols and assisting with the interpretation of findings. This strategy was fundamental to the stated objectives of the research management team of empowering the community and ensuring the cultural safety of the materials and methods. 

SC members were recruited by personal invitation through networks of the research management team. There were nine First Australian members in total: one Aboriginal Elder, all five of the Advanced Health Workers (AHW) covering the geographical area (four of whom identified as Aboriginal and one who identified as both Aboriginal and Torres Strait Islander), an Aboriginal Liaison Officer and two consumers. The input of the AHWs, who worked within the public health system, was particularly sought due to their own perceived role in obesity management [29]. The consumers were a mother and her son, who identified as both Aboriginal and Torres Strait Islander and had received weight management services in the past. Their time and travel costs to attend meetings were paid by the project. Two non-Indigenous members of the research management team, an academic and a health worker, convened and chaired the meetings.

The SC met every 2–3 months, face-to-face in a Queensland Health building, with email communication between meetings. Each meeting comprised 90 min of business discussion, followed by a meal funded by the project and provided by an Indigenous catering company. The meetings were not audio-recorded to allow for frank discussion, but discussion points and decisions were minuted by a member of the research management team. The first meeting was dedicated to relationship building, hearing the lived experience of the consumers, establishing whether there was a need for the research and exploring the research aims. Terms of Reference previously drafted by the research team were negotiated by the entire group, and ongoing interest in SC membership discussed. At the end of the meeting, the agenda and date for the next meeting were set. Subsequent meetings focused on the development of the formative research methods, protocols and materials. Emailed communications included SC meeting minutes, a purpose-developed newsletter and any materials that required comment.

### 2.3. Culturally Tailored Data Collection Materials and Methods

*Data collection protocol—topic guide*: The main consultation instrument was an interview topic guide developed with the SC using an iterative process over several months. The guide documented the key questions to be asked of the First Australian community members about whether weight was an issue in young people and their families. The topics were then written into ‘talking points’, designed to be delivered in a yarning circle by trained interviewers. Yarning circles are recognized as an appropriate method for conducting research with the First Australian community [32]. The topic guide is provided as Supplementary Material File S1. 

*Training package for research data collection interviewers:* A training package was purpose-designed to develop the data collection skills of First Australian health workers and community members. The aim of the training was to discuss how to honor a familiar Indigenous research method (yarning circles) while obtaining qualitative data for analysis. The non-Indigenous university researchers who were experienced in qualitative research and in delivering community-level education worked with the SC to design culturally safe training materials over a two-month period through several rounds of iterative feedback. The training package comprised a slide presentation and written materials, including recruitment posters and the topic guide. 

One of the AHWs on the SC volunteered to undertake a pilot test of the training session to assess the cultural safety of the delivery and to provide process evaluation feedback. The training was delivered by a researcher and observed by another researcher. The feedback from the participant and the observer was incorporated into subsequent training sessions. Participants were trained in how to use the topic guide and talking points and encouraged to adapt the talking points into their own words when conducting their yarning circles. Participants who attended the half-day training sessions received a certificate of completion to document their enhanced skills as research interviewers. Participants either attended the training as part of their paid work or were paid for their time from the project budget, including travel time. 

**Figure 2:**
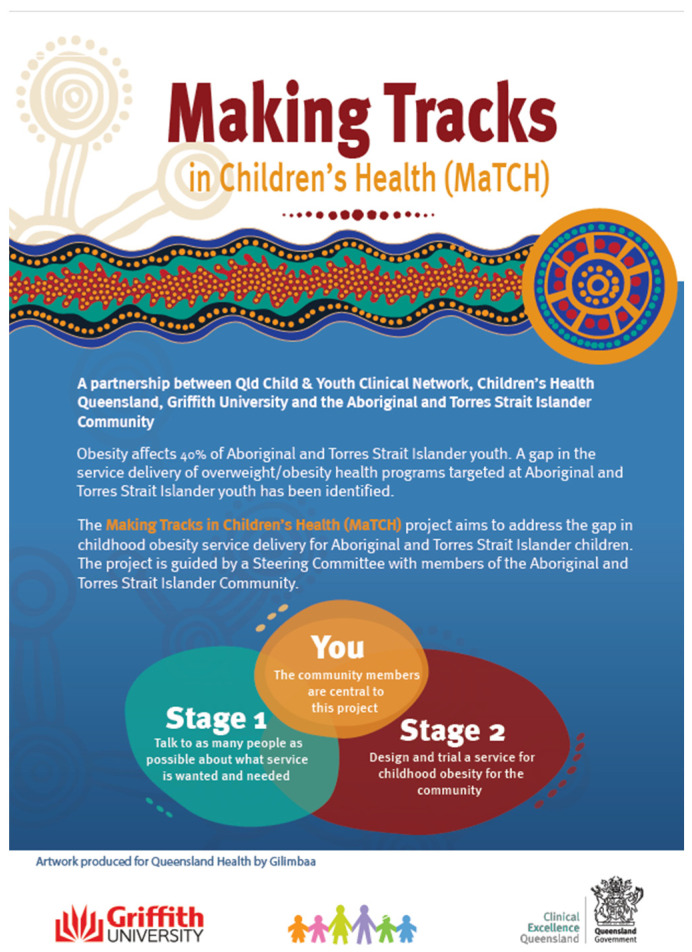
Recruitment material used in the needs assessment for a First Nations pediatric weight-management intervention.

*Promotional and recruitment materials:* An Aboriginal and Torres Strait Islander graphic design service within Queensland Health was engaged to design all print and electronic materials for the SC and the public. This included a banner with Aboriginal and Torres Strait Islander artwork. An example of a poster about the project, including the banner, is shown as Figure 2. 

*Community consultation:* Each trained interviewer was responsible for organizing and conducting at least one yarning circle. They were encouraged to use their own professional and personal networks to liaise with existing community groups to recruit 5–8 community participants interested in the issue of weight in First Australian children. Purpose-developed promotional materials were made available to assist with this task. The research management team members were available to support the yarning circle facilitators in the recruitment process and to organize and pay the costs of catering, venue hire and participant gifts. 

**Figure 3:**
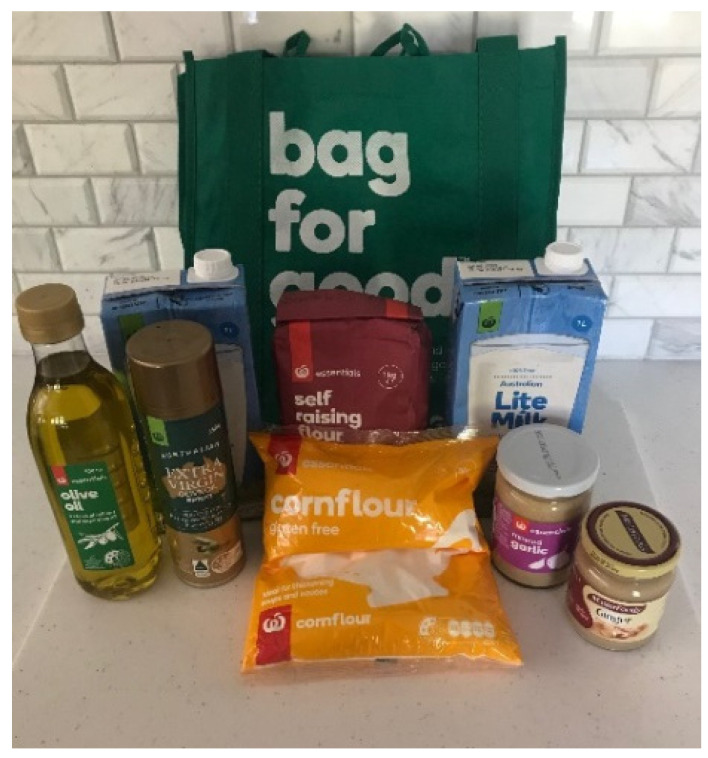
Recipe ingredients gifted to community members participating in the consultation.

Signed, written consent and demographic details were obtained from participants at the start of the yarning circle. An Aboriginal research assistant and a non-Indigenous researcher were available to support the main interviewer in conducting the yarning circle sessions by obtaining informed consent and audio-recording the session for later transcription. The topic guide with the talking points guided the data collection. All participants were given the opportunity to respond to questions, with prompting by the yarning circle interviewer as needed. Lunch was provided as part of the event. Yarning circle participants received a gift of a culturally appropriate recipe book [33] together with a grocery bag worth AUD 20 providing the recipe ingredients as reimbursement for their time (see Figure 3 below). The session was transcribed by the Aboriginal research assistant, and individual scripts were mailed or emailed to participants (according to their stated preference) for verification and the opportunity to add any further comments. Audio recordings and notes taken during the yarning circles were stored securely in a university-owned storage cloud, in line with ethical requirements. 

After the COVID-19 pandemic prevented the use of face-to-face yarning circles, data were collected as individual interviews conducted by the Aboriginal research assistant via telephone. Caregivers of children who had used existing weight management services in the greater Brisbane area were contacted and invited to participate in interviews. The topic guide was amended for individuals to obtain their lived experiences of receiving weight management services, but the other processes of recording and transcription were identical. Individual interview participants were provided with the opportunity to edit or add to their transcript, and they received the recipe book and ingredients package by collecting it from their local community center or through parcel post. 

*Data analysis:* The Aboriginal research assistant transcribed recordings of the yarning circle and individual interviews verbatim. NVivo^®^ software (version 12, QSR International©, Melbourne, Australia) was used to assist the data analysis, which was conducted independently by two members of the research team, one of whom identified as Aboriginal, following Braun and Clarke’s thematic approach [34]. Researchers immersed themselves in the data and independently coded transcripts. Once independent coding had occurred, codes were discussed, collated and clustered to develop themes. All members of the research management team agreed upon the final coding and development of themes. 

### 2.4. Research Team Reflections and Learnings

The research team reflected on the community engagement techniques and culturally tailored data collection methods applied in the research and categorized each according to the objective addressed, the tailoring strategies used (peripheral, evidential, linguistic, constituent-involving, sociocultural) [35] and the extent to which they were implemented as ‘surface’ or ‘deep’ according to the method of Resnicow and colleagues [30]. The team further reflected on the advantages and challenges associated with each technique.

## 3. Results

### 3.1. Study Design

The research commenced in July 2018 after obtaining initial ethical approval from both organizations involved in the research. However, for the reasons described below, the data collection with the First Australian community was not concluded until February 2021. Several requests for extensions to the project period were approved by the grant body, but no additional funds were sought.

### 3.2. Community Engagement Guiding Formative Research Design: The Steering Committee

The SC met face-to-face every three months, commencing in November 2018, with excellent engagement and attendance (at least seven of the nine First Australian members) at the first three meetings. At the first meeting, all the members agreed that weight management was an important priority for children and young people in their community, and all the attendees expressed an interest in continuing their SC role. The group worked well to develop culturally safe materials and processes for data collection. However, the attendance at meetings four and five was low due to illness and role changes among the AHW staff. Additional communications were conducted via email to achieve group consensus. Face-to-face meetings were necessarily halted during 2020 due to the COVID-19 pandemic, with communication maintained via a purpose-developed newsletter written by the research management team and distributed via email. This mode of engagement required more time and achieved less consensus, and the emerging research findings were reported to the SC rather than being discussed. In 2021, when the data collection was complete, the group was thanked for their participation and disbanded. 

### 3.3. Culturally Tailored Data Collection Materials and Methods

Four AHWs and an adult community representative completed one of the three half-day interviewer training sessions by October 2019. After completing their training, they used recruitment materials to establish times for yarning circles with community groups. One trained interviewer planned a yarning circle with the Elders in their geographical area; however, this was cancelled due to ‘sorry business’. Two other trained interviewers commenced extended leave from work for health reasons and were not able to organize any yarning circles.

One AHW successfully organized and held a yarning circle consultation with a ‘Mums and Bubs’ group (seven participants) in a community center in November 2019. She had organized to conduct other yarning circles in different geographical locations after the Christmas period. In the meantime, COVID-19 emerged in Queensland, and the government declared a state of emergency in late January 2020 [36]. For the health and safety of the AHW and community participants, these yarning circle consultations were initially postponed then abandoned due to continued government-imposed lockdowns. It was not feasible to reinitiate this method given that community organizations remained closed or ceased holding their usual group activities. While data collection continued, it took the form of one-to-one interviews conducted by telephone by the Aboriginal research assistant. This was a more resource-intensive method of data collection than yarning circles and focused on members of the community who had previously received outpatient or primary care weight management services, which limited participant heterogeneity.

### 3.4. Research Team Reflection and Learnings

The First Australian participation in the formative research is summarized in Table 1. Each of the major components of the research is described according to which of the three stated research team objectives (empowerment, research capacity and cultural safety) was addressed, the intended purpose of the strategy and the strategies used to enhance community participation. Each component is also evaluated according to categorization [35], the level of cultural tailoring [30] and the advantages and challenges perceived by the research management team. In our assessment, most of the project components achieved the deep sociocultural level that was being aimed for, despite the limitations noted.

## 4. Discussion 

The study described in this paper used a CPAR approach in designing the materials and methods for formative research for a weight-related nutrition intervention with First Australian children. The results of the data collection and analysis, which will be reported separately, will inform the next phase of the project—the co-design of a service within a particular setting relevant to the First Australian community. The current paper describes our attempt as non-Indigenous researchers to deeply and authentically involve a First Australian community in the research process and reflects on the challenges that arose. This paper adds to the emerging literature on detailed methods for conducting culturally safe, formative research for interventions aimed at addressing the nutritional health needs of Indigenous communities. 

The CPAR approach was useful for guiding the research management team in attempting what the Resnicow framework [30] refers to as a ‘deeper’ approach to cultural adaptation in this formative research design. Other authors have found participatory approaches to research with First Australians to be effective [37,38,39], but not always applied in the formative phase of the intervention research [40]. In a paper published in 2022, Beks and colleagues describe how they used a participatory research approach in conducting yarning circles with First Australians about chronic disease [37]. They used the CONSIDER statement to reflect on the process and found their approach to be ethical and able to generate research relevant for the community [37]. Miller and colleagues, in a study on the influenza pandemic in First Australian communities, found PAR to be a useful approach for the collection of qualitative data from the community [38]. Both research teams noted several challenges in using the approach, including the need for flexibility and adaptability on the part of researchers [37,38]. Despite being experienced researchers, the research management team were on a steep learning curve throughout the current project. We needed to learn to be flexible in reconsidering planned processes and adapting to personnel changes, not to mention the extreme challenges of the pandemic. The ways in which we tried, and sometimes failed, to be flexible and adapt are described below. 

The establishment of the SC was the key community engagement strategy, and this technique worked very well until the capacity of some members was exhausted. Canuto and colleagues used a similar structure, which they called a Local Advisory Committee, to advise on the feasibility of the project methods and ensure cultural appropriateness [31]; however, they make no further comment on the effectiveness of this group in their methodology or results paper [41]. Sharmil and colleagues established an ‘Aboriginal Working Party’ as co-researchers on their project [39]. Their group of 10 met bi-monthly to share information, problem solve and network about a research project on drug and alcohol and mental health comorbidity [39]. We deliberately used the term ‘Steering Committee’ to encourage a high degree of ownership of the research project. Despite this title, there was some initial confusion about the role of the SC, with some members seeing the SC as a community consultation on the research topic rather than a committee responsible for governance and cultural safety of the research. The non-Indigenous researchers on the SC needed to learn the benefit of revisiting topics multiple times to ensure shared understanding was achieved. The SC tasks of directing research methods and developing culturally safe materials were met; however the intended task of contextualizing the interpretation of the findings was not possible due to the pandemic-induced cessation of face-to-face meetings, followed by the SC being disbanded. As McDonald has noted, one of the challenges of PAR is the community members being able to maintain their commitment over time [25]. This is not surprising given the competing priorities of social responsibilities and challenges faced by members of this population group, making a research collaboration more difficult to commit to in the long term. Another issue was the expectation by the research management team that the SC would be able to achieve consensus as a group rather than expressing varied perspectives as individuals.

Collecting data using yarning circles was one of the strengths of this project. The suggestion to use yarning circles arose from the SC, who co-developed the talking points for the protocol, and several SC members completed the data collection training. Yarning circles were designed to encourage community participants to speak freely in a format with which they were familiar and in a comfortable environment. The training was designed to build workforce research capacity, a strategy that Gwynne and colleagues found to be missing from most of the 26 nutrition interventions with First Australians included in their systematic review [19]. The research management team in the current project attempted to set aside some of their ‘power’ as research ‘experts’ in embracing an unfamiliar method and supporting the First Australian researchers in leading the data collection. This flexible approach was worthwhile given that the yarning circle was a highlight of the entire research project. The AHW conducting the yarning circle was a talented facilitator who led a rich discussion, and she was willing and able to conduct more sessions. Unfortunately, the pandemic meant that the method was adapted to individual interviews. While the individual interview data were relevant, they lacked the richness of the yarning circle data, and the loss of community organizations as a source of recruitment is likely to have decreased the diversity of views. 

As other teams have also reported, the research process was challenged by personnel changes in both the health sector and on the research management team [37]. Of the five SC members provided with data collection training, only one AHW remained available to conduct yarning circles before they were ceased due to the pandemic. By the time social distancing rules eased, that AHW had a new full-time position and was no longer available to collect data. This highlights the issue of the scarcity of qualified First Australian health workers, with several AHW positions vacated during the course of the project. The extended duration of the project also resulted in staffing changes on the research team, as one of the chief investigators left the health sector for a new position and an academic researcher commenced 12 months of parental leave. 

In their scoping review of the effects of nutritional interventions on cardiovascular health outcomes in First Australian communities, Porykali and colleagues found the most successful projects were those of the longest duration [20]. The short-term nature of the funding was an issue in our project. The initial term for the project was 18 months, from mid-2018 to the end of 2019, which was intended to cover everything from partnership formation to a health economic evaluation of an intervention trial. The research management team had planned for the formative research to take six months; however, by the end of 18 months, only one formative research yarning circle had been conducted. While delays to the study timeline were exacerbated by the COVID-19 pandemic, there were several other factors at play. Sharmil and colleagues found that just the process of obtaining ethical clearance for their project took 18 months [39]. Working with First Australians requires extensive investment in building relationships before anything can be achieved; however, this takes time, which takes money. While the project budget paid the First Australians for their time, it did not cover the significant time investment in relationship building by the research management team, which was absorbed by their employing institutions. Another strategy found to be effective is for professionals already partnering with First Australian organizations and communities to build on those existing relationships in conducting research [41]. While we were fortunate in obtaining multiple timeline extensions from the funding agency, no additional funds were made available.

This emphasizes the need for a different approach to research with the First Australian community proposed by Wilson and colleagues, who argue for a change to the narrative away from a ‘deficits approach’ to a ‘strengths approach’, privileging the richness of their traditional foodways [42]. Wilson and colleagues further call for a decolonization of nutrition research with this community, requiring a shift on the part of researchers and funding agencies [42]. We add our voice to those of other researchers in arguing that funding agencies and government bodies should allocate resources commensurate with the time and effort needed for researchers to decolonize their approach and form authentic partnerships to address the needs of Indigenous communities [39,42]. However, we acknowledge that this will require a significant shift in the current system that rewards academics for achieving large research grants with well-defined research protocols and for publishing highly cited papers [23], actions that are possibly the antithesis of achieving authentic engagement and co-producing research methods to maximize community impact. 

A personal shift on the part of researchers who may not have previously questioned their white privilege would also be of benefit. As academics and health professionals trained in Western research methods and accustomed to managing projects on tight timeframes to achieve stated objectives, attempts at decolonizing were confronting. Reflection and retraining in Indigenous research methods helped us learn to be more flexible and to develop patience with shifts in priorities and timelines. Since this project commenced, several resources have been developed to help researchers conduct culturally safe and relevant research, such as the Aboriginal and Torres Strait Islander Quality Appraisal Tool to evaluate research [43] and tools and resources from the Lowitja Institute [44], and these should be used to guide future research.

As for all research projects, together with the strengths, we acknowledge the limitations. This work was conducted with Aboriginal and Torres Strait Islander people in one geographical area of Queensland, and the results were not intended to be generalized beyond this group. While we surmised that personal and professional circumstances kept members away from SC meetings as the project progressed, there may have been some other reason for their decreased engagement. Process evaluation interviews or surveys with the SC members would have been useful to ascertain the reasons for non-attendance. This was not undertaken in case it added to whatever burden had prevented their attendance. While it would have been desirable to have a First Australian dietitian on the research management team, at the time of project commencement, there were only 37 First Australians who had ever achieved this qualification nationally. We were fortunate to be able to hire an Aboriginal public health nutritionist with an interest in nutrition as a research assistant. 

## 5. Conclusions

The application of CPAR to this formative research involved First Australians in the research design and development of culturally appropriate research materials and methods. Key to the success of the formative research was the commitment of the SC (especially initially) and the dedication and capacity of one AHW who championed the research. The process was time-consuming and challenging, especially in the context of the pandemic. However, the research management team learned to be flexible and to adapt to different ways of researching in an attempt to decolonize research and build cultural competence. The process of conducting formative research builds trust and collaboration, which is foundational for the subsequent implementation phase of the intervention [16]. The aim for the remainder of the project is to keep the First Australian community authentically involved as decision makers through the planning, implementation and evaluation phases of this weight management intervention.

## Figures and Tables

**Table 1:** Community participation and culturally tailored aspects of data collection of felt needs in formative research.

Component	Stated Objectives	Intended Purpose	Strategies to Enhance Community Participation	Category ^1^; Level ^2^	Advantages: What Worked Well	Challenges: What Did Not Work Well
Steering Committee (SC)	(1) Empower the community (3) Ensure cultural safety	Provide lived experience and other expertise in the direction of the research and help develop culturally safe research materials	The SC comprised primarily First Australians (9 of 11 members) SC meetings held face-to-face in familiar and central location Community members reimbursed for their time to attend meetings and provided with cab vouchers to pay for their travel Lunch provided at each meeting, prepared by Indigenous catering company The SC reviewed and advised on research materials at meetings or via email between meetings Newsletters updated progress between meetings	Sociocultural; Deep	SC members drew on their work or lived experience to make a rich contribution to research direction SC members had prior experience working with non-Indigenous health professionals Willingness to share insights into what would/would not work Lunch provided by a catering company liked by SC members Taking time to chat over lunch helped build relationships	Non-Indigenous chairs set meeting agendas, which may have limited discussion scope Consensus decision not possible for all issues, particularly via email Some initial misunderstanding of the scope and role of the SC Significant time commitment required of First Australian SC members The SC was required for more than 12 months, which was not possible for some members
Data collection protocol-topic guide	(1) Empower the community (3) Ensure cultural safety	Support consistency of data collection	The SC advised that data should be collected in yarning circles, and the topic guide protocol should be in the form of ‘Talking points’. Talking points drafted by research team in collaboration with the SC, who assessed cultural safety and relevance. Interviewers were encouraged to put the talking points into their own words.	Sociocultural; Deep	Yarning circles recognized as an authentic Indigenous research technique	Time taken to draft the protocol and have it approved
Training package for research data collection interviewers	(2) Develop research capacity	Support consistency of data collection and upskill First Australian researchers	First Australian interviewers trained in how to use a culturally appropriate research technique (yarning circles) to collect qualitative data. Training package pilot tested by SC member to assess cultural safety before use. Training delivered in a community center located in an Aboriginal community. Lunch provided, prepared by Indigenous catering company. Participants provided with university certificate of completion. Paid for training hours (unless completed in paid work hours).	Constituent-involving; Deep	Training built on the fact that SC members were already comfortable conducting a yarning circle. Training style interactive with time for discussion and questions. Group training sessions allowed colleagues to network over lunch.	Trainers were non-Indigenous research team members Half day plus travel required for participants to attend. Significant time required to find a venue and catering service that everyone was comfortable with
Project promotional and recruitment materials	(3) Ensure cultural safety	Communicate with the public and recruit participants through community agencies	Indigenous artwork provided by Queensland Health for all project materials. SC assessed as culturally safe. Used as posters around community centers to familiarize the public with the project.	Peripheral: Visual; Surface	Written communications were colorful, professionally designed and consistent.	Materials received in pdf format restricted ability to edit. Requests to edit took at least a week.
Interviews with First Australian community members	(1) Empower the community (2) Develop research capacity (3) Ensure cultural safety	Seek the lived experiences and perceptions of community members on weight management in children	First Australians trained and paid to conduct yarning circle interviews. Research assistants available to support sessions if requested. Yarning circles held in venue familiar to the community. Food provided at the session. Materials to support data collection provided in hard copy. All yarning circle and interview participants were provided with a cookbook and a shopping bag of groceries with the ingredients required to make recipes from the cookbook. Participants provided with the opportunity to edit or add to their transcript.	Sociocultural; Deep	Great attendance at Yarning circle. Participants familiar and comfortable with this way of communicating. Interviews were all conducted by the Aboriginal research assistant. All participants agreed to being audio recorded. The recipe books and ingredients were carefully chosen by a female Aboriginal researcher, and people appreciated the gesture.	Yarning circles abandoned due to pandemic; breadth of community views lost. Loss of trained interviewers due to changed work role in pandemic. Individual interviews conducted by phone, which may have restricted the richness of responses.
Data analysis and interpretation	(2) Develop research capacity	To draw conclusions from the data to inform intervention development	Female Aboriginal member of research team assisted with analysis and interpretation of the qualitative data.	Constituent-involving; Deep	The Aboriginal researcher brought cultural understanding to the analysis. She worked well with the non-Indigenous RA in coding.	Consultation findings were reported back to the SC rather than their interpretations discussed due to cessation of face-to-face SC meetings.

**^1^** Categorization of the tailoring strategy according to Kreuter et al., 2003 [35]. ^2^ Level of tailoring strategy categorized as surface/deep according to the method of Resnicow et al., 1999 [30].

## Data Availability

The data presented in this study are available on request from the corresponding author. The data are not publicly available due to ethical restrictions.

## Supplementary Materials

The following supporting information can be downloaded at https://www.mdpi.com/article/10.3390/nu15234982/s1: Supplementary File S1. Topic guide (Talking points).
