# Supplementary material for: How Can We Find Out What Indigenous Children and Their Families Need to Manage Weight? Lessons from Formative Nutrition Intervention Research with First Australians
Source: Nutrients. 2023 Dec 1;15(23):4982. doi: 10.3390/nu15234982 (PMC10708399; doi:10.3390/nu15234982)
Supplement: Supplementary file 1 [file nutrients-15-04982-s001.zip › nutrients-2706439-supplementary.pdf]

Supplementary Material File S1: Talking Points (Tables 1 to 4)

1. Need (Is there a need for a nutrition-related health service for childhood weight?)

Question	Prompts
**Icebreaker Q:** Before we get started, let's get to know each other a little bit. If everyone can share their name and something about themselves that would be great! (For eg. What brought you here today? Are you a mother, father, grandmother? Do you have kids/grandkids? How many?)	
Do you think overweight and obesity is an issue in Aboriginal & Torres Strait Islander children in your community?	- If yes, why do you say that?<br>- If no, why do you say that?
Do you think families would like help and advice to talk about and address the issue?	- How should this advice be offered?
Would healthy foods for the Aboriginal & Torres Strait Islander community help?	- Why or why not?
Would you like someone to talk to you about healthy food and your child's health?	- Who should talk to them about this?
Are there any reasons why children/families would or wouldn't use a healthy eating service to help healthy weight in children in your community?	
What have been the barriers for children/families to using a nutrition-related health service previously?	
**Other feedback on need for a new service questions:**	

2. Service Design (what should the service look like?)

Question	Prompts
If the service looked like this (show Model of Service Delivery), what would you think? (Eg. A local service tailored for Aboriginal & Torres Strait Islanders, that provides dietitian and exercise services, and nutrition support)	- What do you like? Dislike?<br>- Anything missing? Anything to remove?
What do you think the service should look like?	- Who should attend?<br>- Should they be individual or group appointments?
Who should help with a healthy food service (i.e. Health Worker, Dietitian, Exercise Physiologist, Nurse, Doctor, Spiritual Healer)?	
How often to you think children/families should visit the service?	
Where do you think the service should be located?	- Within existing service or a new place?<br>- Do you have any suggestions where it should be located?
**Other feedback on questions for what the new service should look like:**	

3. Content (what should be talked about?)

Question	Prompts
What areas of health do you think the clinic should focus on to support a healthy weight in children?	- What about food and nutrition?<br>o Traditional foods?<br>- Physical activity?<br>- Screen-time?<br>- Social and emotional wellbeing?
**Other feedback on questions related to what should be talked about:**	

4. Engagement (connecting with the new service)

Question	Prompts
How would you/families like to find out about a new nutrition-related health service to help with healthy weight in children in your community?	
How would you/families like trust to be built with the new service and with Health Professionals delivering the service?	
What would make you/families want to come back for further sessions at the service?	
Can you suggest a name for the service?	- What social media outlets should we use to get more input?
**Other feedback on connecting with the new service questions**	
